# Implantation of a novel insertable cardiac monitor: preliminary multicenter experience in Europe

**DOI:** 10.1007/s10840-024-01821-y

**Published:** 2024-05-16

**Authors:** S. Fareh, S. Nardi, L. Argenziano, A. Diamante, F. Scala, C. Mandurino, M. Magnocavallo, L. Poggio, M. Scarano, D. Gianfrancesco, F. Palma, M. S. Silvetti, D. Porcelli, M. Racheli, M. Montoy, P. Charles, M. Campari, S. Valsecchi, C. Lavalle

**Affiliations:** 1 Department of Cardiology, Hôpital de La Croix Rousse Et Hôpital Lyon Sud, Hospices Civils de Lyon, 103 Gd Rue de La Croix-Rousse, 69004 Lyon, France; 2 Pineta Grande” Hospital, Castel Volturno, CE Italy; 3 Clinica Sanatrix, Naples, Italy; 4 Casa Di Cura “Villa Azzurra”, Siracusa, Italy; 5 Fatebenefratelli Hospital, Naples, Italy; 6 Santissima Annunziata” Hospital, Taranto, Italy; 7 Ospedale Isola Tiberina – Gemelli Isola, Rome, Italy; 8 Ospedale Maggiore Di Lodi, Lodi, Italy; 9 Madonna del Soccorso” Hospital, San Benedetto del Tronto (AP), Italy; 10 “L. Bonomo” Hospital, Andria, Italy; 11 Mons. Dimiccoli” Hospital, Barletta, Italy; 12 Bambin Gesù” Pediatric Hospital, Rome, Italy; 13 San Pietro-Fatebenefratelli Hospital, Rome, Italy; 14 San Pellegrino Hospital, Castiglione Delle Stiviere (MN), Italy; 15 Boston Scientific Italia, Milan, Italy; 16 Department of Cardiovascular, Respiratory, NephrologicalAnesthesiological and Geriatric Sciences, “Sapienza” University of Rome, Policlinico Umberto I, Rome, Italy

**Keywords:** Insertable cardiac monitor, Implantation, Loop recorder, Arrhythmias, Syncope, Atrial fibrillation, Cryptogenic stroke

## Abstract

**Background:**

The LUX-Dx™ is a novel insertable cardiac monitor (ICM) introduced into the European market since October 2022.

**Purpose:**

The aim of this investigation was to provide a comprehensive description of the ICM implantation experience in Europe during its initial year of commercial use.

**Methods:**

The system comprises an incision tool and a single-piece insertion tool pre-loaded with the small ICM. The implantation procedure involves incision, creation of a device pocket, insertion of the ICM, verification of sensing, and incision closure. Patients receive a mobile device with a preloaded App, connecting to their ICM and transmitting data to the management system. Data collected at European centers were analyzed at the time of implantation and before patient discharge.

**Results:**

A total of 368 implantation procedures were conducted across 23 centers. Syncope (235, 64%) and cryptogenic stroke (34, 9%) were the most frequent indications for ICM. Most procedures (338, 92%) were performed in electrophysiology laboratories. All ICMs were successfully implanted in the left parasternal region, oriented at 45° in 323 (88%) patients. Repositioning was necessary after sensing verification in 9 (2%) patients. No procedural complications were reported, with a median time from skin incision to suture of 4 min (25th–75th percentiles 2–7). At implantation, the mean R-wave amplitude was 0.39 ± 0.30 mV and the P-wave visibility was 91 ± 20%. Sensing parameters remained stable until pre-discharge and were not influenced by patient characteristics or indications. Procedural times were fast, exhibited consistency across patient groups, and improved after an initial experience with the system. Operator Operator feedback on the system was positive. Patients reported very good ease of use of the App and low levels of discomfort after implantation.

**Conclusions:**

LUX-Dx™ implantation appears efficient and straightforward, with favorable post-implantation sensing values and associated with positive feedback from operators and patients.

**Graphical Abstract:**

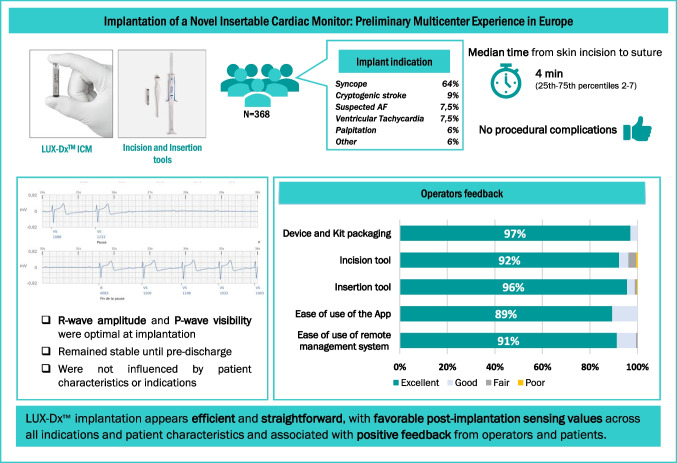

**Supplementary Information:**

The online version contains supplementary material available at 10.1007/s10840-024-01821-y.

## Introduction

Insertable cardiac monitors (ICMs) serve as crucial tools for the long-term monitoring of patients with known or suspected cardiac arrhythmias. Over time, the indications for ICM usage have broadened [1–3], reflecting advancements in device miniaturization, simplification of subcutaneous insertion procedures, enhancement of arrhythmia detection algorithms, and the incorporation of novel functionalities [4]. The LUX-Dx™ (Boston Scientific, Marlborough, MA, USA) is a novel ICM, incorporating dual-stage arrhythmia detection algorithms and remote programming capabilities. While prior studies have examined the remote programming of this device within prospective and real-world settings [4, 5], there remains a paucity of literature exploring the implantation experience of the LUX-Dx™ ICM, particularly in European contexts. Therefore, the aim of this investigation was to provide a comprehensive description of the LUX-Dx™ implantation experience in Europe during its initial year of commercial use.

## Methods

### Study design

From October 2022 to February 2024, consecutive patients undergoing implantation of a LUX-Dx™ ICM were included at 23 European centers (see Appendix). The decision to implant the ICM was at the discretion of the operator, with no predetermined indication agreed upon among the participating centers. Devices were implanted and programmed according to the local clinical practice. Operators at the centers were requested to collect data and complete a questionnaire to measure satisfaction and provide feedback on the implantation procedure and the system. They evaluated R-wave amplitudes and the visibility of P-waves, defined as the ratio of clearly identifiable P-waves to heart cycles during a 10-s ECG with regular 1:1 conduction. Data were collected at the time of implantation and before patient discharge. An anonymous patient survey collected data and information concerning patients’ pain, paresthesia, and confidence in using the system. As this was a retrospective analysis of anonymized data from a registry in real-life practice, the study was exempt from review and approval by institutional review boards of participating institutions. Postprocessing was conducted in accordance with the European General Data Protection Regulation (UE 2016/679). All data were de-identified to ensure the protection of personal health data, as mandated by European regulations. Patients had granted written approval to contribute data at the time of remote monitoring activation. This study was independent and not funded by industry.

### The device

**Fig. 1:**
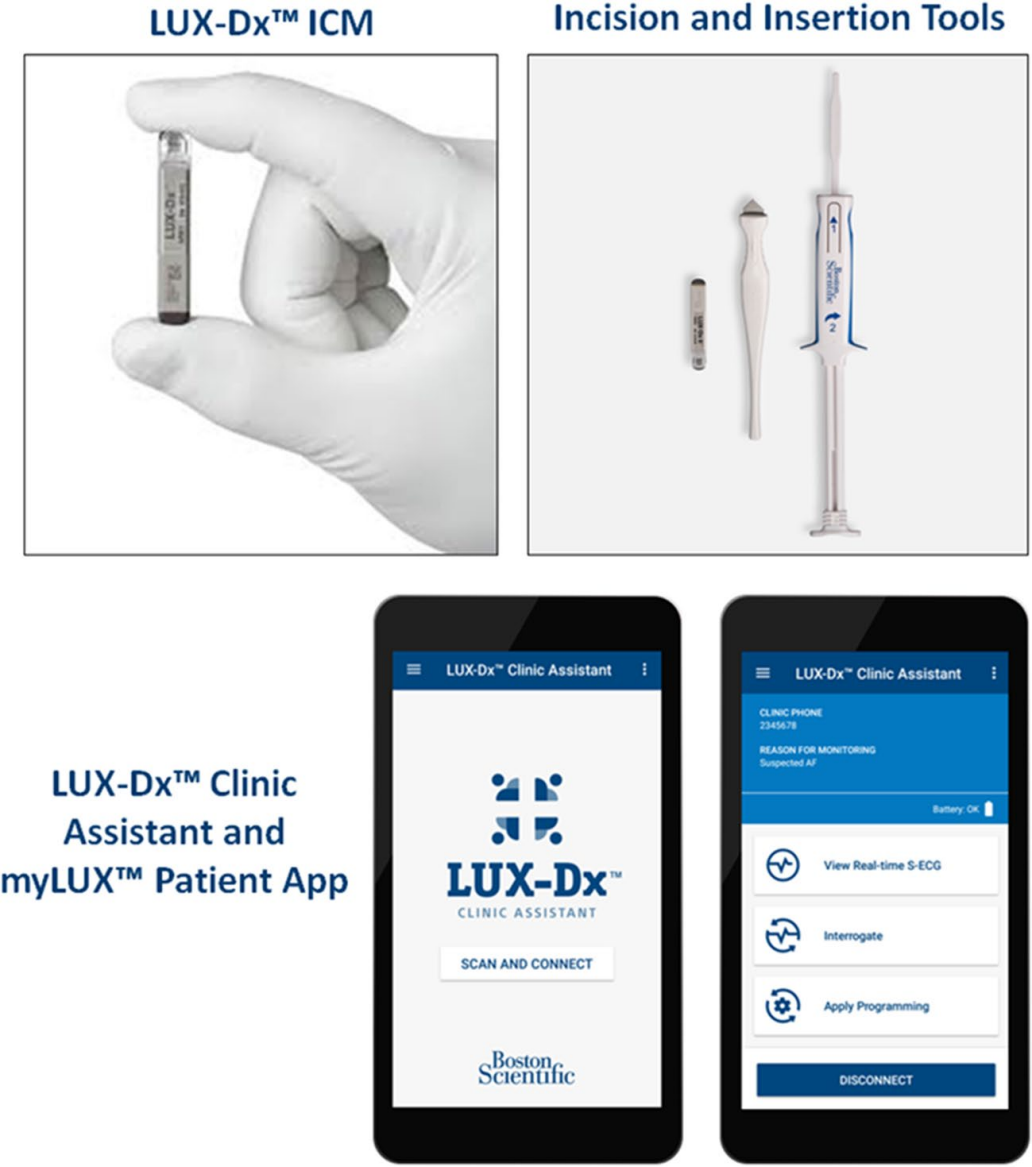
The LUX-Dx™ implantable cardiac monitor (ICM). The system comprises an incision tool and a single-piece insertion tool pre-loaded with the ICM. The LUX-Dx™ Clinic Assistant App allows the operator to connect to the ICM. The myLUX™ Patient App activates the implanted device and transmits data between the ICM and the LATITUDE server

The LUX-Dx™ is a small (1.2 cm^3^) ICM designed to monitor, record, and store data related to cardiac arrhythmias that fall into five categories: pauses, bradyarrhythmias, tachyarrhythmias, atrial fibrillation, and atrial tachycardia. Examples of subcutaneous ECGs are reported in Supplemental Figures. Each category’s algorithm contains settings that can be tailored according to the patient’s specific clinical indications. The LUX-Dx™ ICM provides remote programming capabilities. The implantation kit includes an incision tool and a single-piece insertion tool pre-loaded with the ICM (Fig. 1). The implantation procedure involves incision, creation of a device pocket, insertion of the ICM, verification of sensing, and closure of the incision. The LUX-Dx™ Clinic Assistant App on a mobile device allows the operator to connect to and interrogate the ICM, view ICM device status and real-time S-ECG, and apply programming changes made in the LATITUDE Clarity™ Data Management System to the ICM device. After the procedure, patients receive a mobile device with the preloaded myLUX™ Patient App, designed to activate the patient’s implanted ICM and transmit data between their ICM and the LATITUDE server. The App also offers other user-friendly features intended to empower patients and increase compliance: it shows the monitoring status and provides instructions to help patients reconnect, allows operators to send messages to patients confirming that their data has been received, allows patients to record their symptoms and activity, and provides educational material.

### Statistical analysis

Quantitative variables are reported as means ± SD if normally distributed, or medians with 25th to 75th percentiles in the case of skewed distribution. Normality of distribution was tested by means of the non-parametric Kolmogorov–Smirnov test. Categorical data were expressed as percentages. Differences between mean data were compared by a *t*-test for Gaussian variables and by Mann–Whitney non-parametric test for non-Gaussian variables. Differences in proportions were compared by means of chi-square analysis or Fisher’s exact test, as appropriate. A *p* value < 0.05 was considered significant for all tests. All statistical analyses were performed by means of R: a language and environment for statistical computing (R Foundation for Statistical Computing, Vienna, Austria).

## Results

### Study population

A total of 368 consecutive implantation procedures were conducted across 23 European centers, from October 2022 to February 2024. Syncope (64%) and cryptogenic stroke (9%) were the most frequent indications for ICM implantation (Table 1). The study group included 10 (3%) pediatric patients (< 21 years).

**Table 1:** Baseline clinical parameters and indications for ICM implantation

Parameter	*n* = 368
Male, *n* (%)	208 (57)
Age, *n* (%)	
< 40 years	43 (12)
40–59 years	93 (25)
60–79 years	162 (44)
≥ 80 years	70 (19)
Body mass index, *n* (%)	
< 18.5 kg/m^2^ (underweight)	45 (12)
18.5–24.9 kg/m^2^ (healthy weight)	210 (57)
25.0–29.9 kg/m^2^ (overweight)	84 (23)
≥ 30 kg/m^2^ (obese)	29 (8)
Reason for monitoring, *n* (%)	
Syncope	235 (64)
Cryptogenic stroke	34 (9)
Suspected atrial fibrillation	27 (7.5)
Ventricular tachycardia	27 (7.5)
Palpitation	23 (6)
Other	22 (6)

### Implantation procedure

**Fig. 2:**
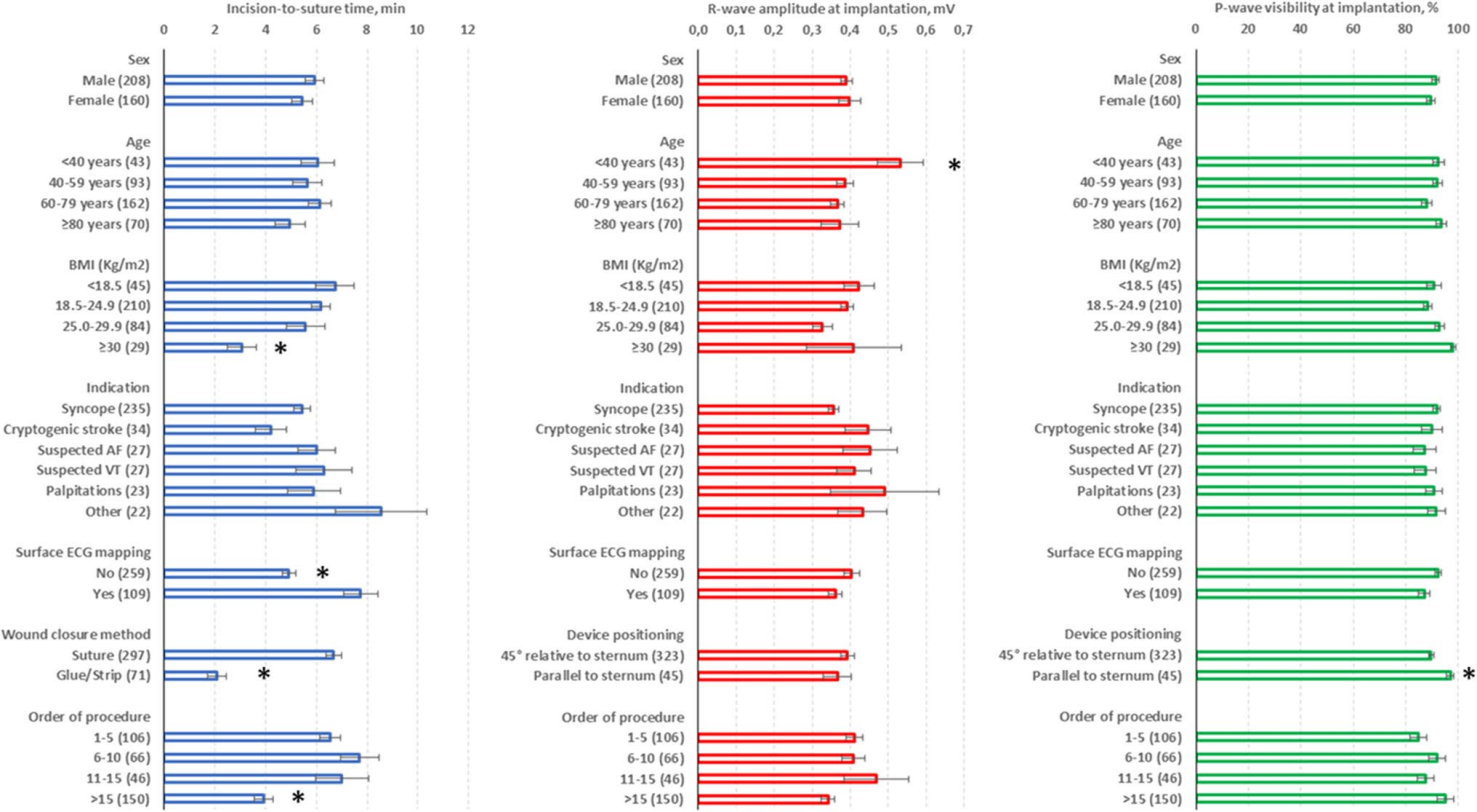
Procedural time, R-wave amplitude, and P-wave visibility at implantation across patient characteristics, indications, and procedural variables (*p < 0.01 versus others)

**Fig. 3:**
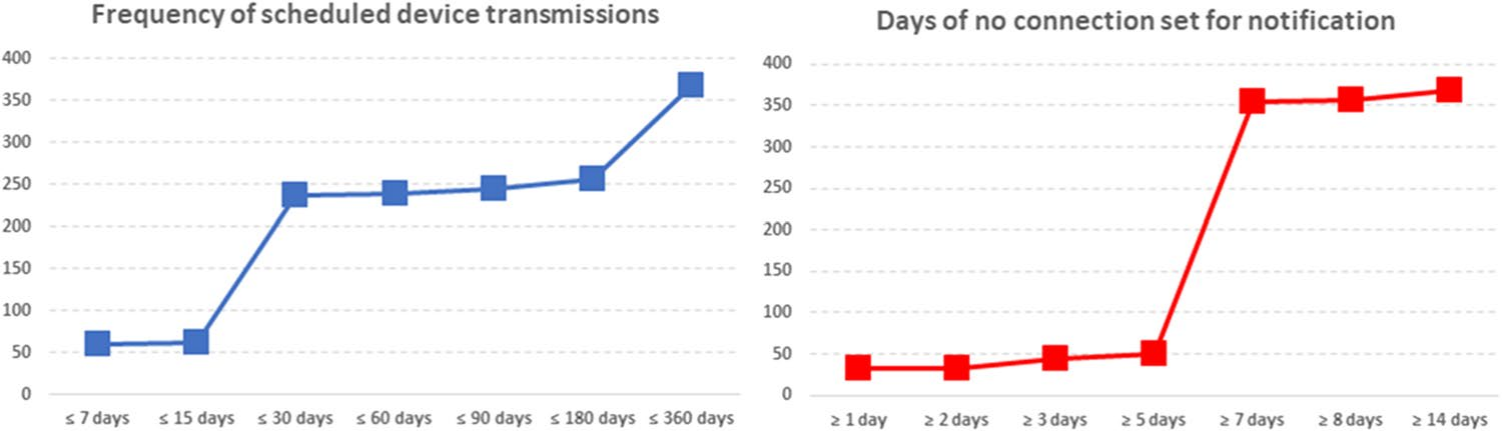
Programmed frequency of scheduled device transmissions and number of days of no connection set for notification

The majority of procedures (92%) were performed in electrophysiology laboratories by experienced operators. Local anesthesia was utilized for all procedures, except for those performed under general anesthesia in 8 pediatric patients. Systemic or local antibiotics were administered before the procedure in 205 (56%) cases (Table 2). Surface ECG mapping to achieve acceptable R-wave amplitude was conducted before 109 (30%) insertions. All ICMs were successfully implanted in the left parasternal region, oriented at 45° in 88% of patients. The median time from skin incision to suture was 4 min (25th–75th percentiles 2–7). Repositioning was necessary after sensing verification in 9 (2%) patients. No procedural complications were reported. Initial connection difficulties were encountered in 2 patients, which were resolved by updating the App in one case and replacing the patient mobile device in the second. The mean R-wave amplitude was 0.39 ± 0.30 mV at implantation and 0.41 ± 0.31 mV before patient discharge (*p* = 0.052). Twenty-four patients did not show sinus rhythm with a regular 1:1 conduction. In the remaining patients, P-wave visibility was 91 ± 20% at implantation and 91 ± 20% before discharge (*p* = 0.790). Comparable implantation durations were observed across various patient characteristics or indications, except for shorter times reported for patients with a higher body mass index, in case of ECG mapping omission or sutureless wound closure, or achieved after the first 15 cases (Fig. 2). No differences in R-wave amplitude and P-wave visibility were observed among subgroups, with the exception of higher R-wave amplitudes in the younger patients and higher P-wave visibility in patients with the device positioned parallel to the sternum (Fig. 2). In the vast majority of devices, the programming of detection parameters was not changed from the nominal setting automatically proposed for the specific reason for monitoring, and the option of recording symptoms by the patient was enabled (Table 2). Scheduled device transmissions were usually programmed at least once every 30 days, and frequently notifications were enabled for losses of connection of at least 7 days (Fig. 3). Survey questions on the overall operator and patient experience with the implantation procedure are reported in Table 3. Most patients did not report any pain or paresthesia either at implantation or at discharge. Two hundred and forty-two (66%) patients were discharged on the same day of the procedure, with the remaining patients discharged after a median of 2 (25th–75th percentile: 1–2) days.

**Table 2:** Implantation procedure

Parameter	*n* = 368
Operator, *n* (%)	
Physician	365 (99)
Nurse	3 (1)
Place of procedure, *n* (%)	
Electrophysiology laboratory	338 (92)
Ambulatory room	30 (8)
Anesthesia, *n* (%)	
Local	360 (98)
General	8 (2)
Antibiotic prophylaxis, *n* (%)	205 (56)
Incision site preparation, *n* (%)	
No	32 (9)
Betadine	280 (76)
Chlorhexidine	56 (15)
Surface ECG mapping, *n* (%)	109 (30)
Incision-to-suture time, min*	4 [2–7]
Incision time	1 [0–1]
Insertion time	1 [0–2]
Verification time (S-ECG evaluation)	1 [0–1]
Suture time	1 [1–3]
Device positioning, *n* (%)	
45° relative to sternum	323 (88)
Parallel to sternum	45 (12)
Wound closure method, *n* (%)	
Suture	297 (81)
Surgical glue	64 (17)
Adhesive strip	7 (2)
Post-procedural antibiotics, *n* (%)	65 (18)
Nominal parameters setting, *n* (%)	364 (99)
Symptoms recording enabled, *n* (%)	360 (98)

*Median [25th–75th percentiles]

**Table 3:** Survey questions on operator and patient experience with the implantation procedure

Operator experience:	Excellent	Good	Fair	Poor	Bad
Device and kit packaging	357 (97)	11 (3)	0 (0)	0 (0)	0 (0)
Incision tool	340 (92)	14 (4)	12 (3)	2 (1)	0 (0)
Insertion tool	352 (96)	12 (3)	3 (1)	1 (0)	0 (0)
Ease of use of the App for sensing verification and ICM activation	329 (89)	39 (11)	0 (0)	0 (0)	0 (0)
Ease of use of remote management system for patient enrollment	336 (91)	30 (8)	2 (1)	0 (0)	0 (0)
Patient experience:	No	Mild	Moderate	Severe	
Pain during implantation	336 (91)	31 (9)	1 (0)	0 (0)	
Paresthesia after implantation	362 (98)	6 (2)	0 (0)	0 (0)	
Pain at discharge	360 (98)	8 (2)	0 (0)	0 (0)	
Paresthesia at discharge	364 (99)	3 (1)	0 (0)	1 (0)	
	Completely	Somewhat	Not at all		
Confidence in using the patient App	327 (89)	31 (8)	10 (3)		

## Discussion

In this study, we present the initial experience of LUX-Dx™ ICM implantation in clinical practice in Europe. The implantation procedure was safe and straightforward and yielded favorable outcomes in terms of system functionality, as well as satisfaction reported by both operators and patients. The patient cohort exhibited diverse clinical characteristics. Consistent with previous observational studies [5–7], the indications for ICM implantation varied, with unexplained syncope being the most common indication, supported by robust evidence and established recommendations [1, 2].

The majority of procedures were conducted by physicians in electrophysiology laboratories, although positive experiences have been reported with procedures performed by nurses and in alternative settings [8–12]. Local anesthesia was used in almost all cases. Antibiotic prophylaxis was administered before the procedure in 56% of cases, consistent with previous literature where prophylaxis rates ranged from 0 to 50% [7, 13, 14]. Surface ECG mapping was conducted before a minority of procedures (30%). The efficacy of the applied anatomically based placement approach was confirmed by the low rate of intraoperative ICM repositioning required after signal verification, consistent with previous studies [1, 15]. Despite being an analysis of initial implantations, procedural times were fast, consistent with, or even shorter than those reported for previous systems (typically ranging from 5 to 9 min) [7, 13, 14, 16]. Procedural times exhibited consistency across patient groups, and shorter values when ECG mapping was omitted or sutureless systems for wound closure were used. After an initial experience with the system, a further reduction in procedural times was also observed. Sensing parameters at implantation were optimal, remained stable until pre-discharge, and were not influenced by patient characteristics or indications, consistent with findings from other ICM studies [17]. R-wave amplitudes were higher in the younger patients, in agreement with previous studies that showed better R-wave sensing in pediatric patients, being the amplitude inversely proportional to the patient body surface area [18]. Furthermore, P-wave visibility was favorable compared to values reported for other systems [19]. Surface ECG mapping did not yield improved sensing parameters, whereas positioning the device parallel to the sternum resulted in slightly enhanced P-wave visibility. This finding has been previously shown with ICMs with long sensing vectors, although significant differences were not detected [20]. The consistency of results across varying indications and with advancing age is reassuring and particularly significant as it has been demonstrated that the utility of ICMs increases with age, with new diagnoses more frequently made and important treatment changes more frequently triggered in older patients [6].

Operator feedback on incision and insertion tools, as well as on the sensing verification App and remote management system for enrollment and programming, was positive. Patients reported very good ease of use of the App, with over 90% not experiencing pain during the procedure and over 98% reporting no pain or paresthesia post-implantation. This contrasts favorably with discomfort reported after implantation of previous ICMs with long sensing vectors (no relevant post-implantation pain in 47% and no sustained paresthesia in 51% of patients) [13].

The implementation of remote monitoring for ICMs presents the challenge of a high volume of transmissions and frequent misdiagnoses [21]. Consequently, there has been an effort to develop improved arrhythmia detection algorithms aimed at reducing false-positive detections [22]. Moreover, there is increasing emphasis on the programming of ICMs, with the recent Expert Consensus Statement on Practical Management of the Remote Device Clinic [23] recommending tailored alert programming based on clinical indications. The LUX-Dx™ ICM automatically customizes detection parameters based on the specific reason for monitoring set at enrollment. This aligns with recommendations to tailor programming, without requiring manual deviation from the nominal parameters set, as observed in the present study. Additionally, symptom recording was frequently enabled in our patients, as also recommended for assessing symptom-rhythm correlation [23]. The guidelines also suggest reprogramming in cases of frequent false positives or nonactionable alerts. Indeed, strategic reprogramming can effectively reduce transmission volumes [8], albeit potentially necessitating additional office visits. In response, remote programming capabilities have been introduced in modern ICMs to alleviate alert burden without the need for in-person consultations, aligning with recommendations that in-office visits are unnecessary for the ongoing care of ICM patients [23]. The real-world use of ICM remote programming has been recently described, reviewing data from more than 8000 patients in the USA with the LUX-Dx™ ICM [5]. The analysis showed that 24% of devices were reprogrammed, with 82% of reprogramming events occurring remotely, mostly within the first 30 days post-implantation, suggesting that remote programming may enhance clinical efficiency and patient care without additional workload. In the present analysis, the device was set to detect connection loss of at least 7 days in almost all patients, with notifications sent to the patient’s mobile device to ensure consistent connectivity. High levels of remote monitoring were previously demonstrated with the LUX-Dx™, minimizing transmission failures and maintaining continuous connectivity throughout the monitoring period [4]. This addresses issues of transmission delays reported with previous systems [24, 25] and is also important to potentially reduce transmission volume. In fact, guidelines allow the elimination of scheduled transmissions in cases of uninterrupted connectivity [23]. However, our study revealed that such scheduled transmissions are still often programmed every 30 days. Therefore, eliminating these transmissions could significantly reduce the overall volume of transmissions.

### Practical implications

In summary, the initial performance of the novel LUX-Dx™ ICM appears promising in terms of ease of implantation, acute electrical performance, and safety across various patient groups. Our preliminary implantation experience suggests that after the first 15 procedures, the implantation time decreases. Additionally, employing sutureless wound closure systems can expedite the procedure. Similarly, omitting ECG mapping, which does not enhance sensing parameters, and instead favoring a device positioning parallel to the sternum, appears to shorten the procedure and improve P-wave visibility. Further data on safety and performance during follow-up are desirable. However, interim results from the LUX‐Dx PERFORM trial indicate a favorable safety profile with few adverse device effects [4].

### Limitations

Our findings may have potential limitations. This study involved a retrospective analysis of clinical data collected prospectively in real-life practice. While the participating centers included patients who consecutively underwent implantation of a LUX-Dx™ ICM, we did not gather data on patients who received implantation of other ICM systems during the observation period. Consequently, we cannot rule out the possibility of selection bias. Furthermore, the qualitative nature of the patient- or operator-reported outcomes may have introduced additional bias.

## Conclusions

LUX-Dx™ implantation appears efficient and straightforward, with favorable post-implantation sensing values across all indications and patient characteristics and associated with positive feedback from operators and patients.

## Electronic supplementary material

Below is the link to the electronic supplementary material.

Supplementary file1 (JPEG 762 KB)

Supplementary file2 (JPEG 982 KB)

Supplementary file3 (JPEG 1006 KB)

## Data Availability

The experimental data used to support the findings of this study are available from the corresponding author upon reasonable request.

## Appendix

List of participating centers

- Hôpital de la Croix Rousse et Hôpital Lyon Sud, Hospices Civils de Lyon, Lyon, France: Thibault Thenard, Samir Fareh, Mathieu Montoy, Paul Charles
- “Pineta Grande” Hospital, Castelvolturno (CE), Italy: Veronica Amato, Stefano Nardi
- Clinica Sanatrix, Naples, Italy: Vittoria Marino, Luigi Argenziano
- Casa di Cura “Villa Azzurra”, Siracusa, Italy: Aldo Centaro, Nicole Monaca, Alessandro Diamante
- Fatebenefratelli Hospital, Naples, Italy: Giuseppe Franzone, Cristina De Colle, Vittoria Miano, Fernando Scala
- “Santissima Annunziata” Hospital, Taranto, Italy: Giovanni Luzi, Michela Scoletta, Cosimo Mandurino
- Ospedale Isola Tiberina—Gemelli Isola, Rome, Italy: Viviana Cordelia, Marco Borzi, Matteo Conte, Michele Magnocavallo
- Ospedale Maggiore di Lodi, Lodi, Italy: Francesco Villella, Luca Poggio
- “Madonna del Soccorso” Hospital, San Benedetto del Tronto (AP), Italy: Sara Mennilli, Michele Scarano
- “L. Bonomo” Hospital, Andria, Italy: Francesco Bartolomucci, Piero Vitti, Gianluca Robles, Domenico Gianfrancesco
- “Mons. Dimiccoli” Hospital, Barletta, Italy: Giuseppe Diaferia, Giuseppe Carpagnano, Francesca Pomarico, Francesco Palma
- “Bambin Gesù” Pediatric Hospital, Rome, Italy: Cristina Raimondo, Fabio Anselmo Saputo, Ilaria Tamburri, Fabrizio Drago, Massismo Stefano Silvetti
- San Pietro-Fatebenefratelli Hospital, Rome, Italy: Barbara Romani, Jacopo Costantino, Daniele Porcelli
- San Pellegrino Hospital, Castiglione delle Stiviere (MN), Italy: Carlo Agostino Oliva, Marco Racheli
- Department of Cardiovascular, Respiratory, Nephrological, Anesthesiological and Geriatric Sciences, “Sapienza” University of Rome, Policlinico Umberto I, Rome, Italy: Nicola Pierucci, Carlo Lavalle
- Ospedale di Lavagna, Lavagna (GE), Italy: Paolo Donateo, Jacopo Senes, Guido Parodi
- “San Poalo” Hospital, Civitavecchia (RM), Italy: Sergio Calcagno, Giovanni Biscotti
- “SS Annunziata” Hospital, Sassari, Italy: Gavino Casu, Dario Argiolas, Graziana Viola
- “Magalini” Hospital, Villafranca (VR), Italy: Gabriele Zanotto
- “Parodi Delfino” Hospital, Colleferro (RM), Italy: Sabina Ficili, Aldo Lacquaniti
- Ospedale Generale Regionale “F. Miulli”, Acquaviva delle Fonti (BA), Italy: Massimo Grimaldi, Vincenzo Caccavo, Luca Sgarra
- “Mater Salutis” Hospital, Legnago (VR), Italy: Davide Sandrini, Emanuela Visentin
- Presidio Ospedaliero di Anzio-Nettuno, Anzio (RM), Italy: Natale Di Belardino, Armando Molinari
