# Supplementary figures and images for: Implantation of a novel insertable cardiac monitor: preliminary multicenter experience in Europe
Source: J Interv Card Electrophysiol. 2024 May 16;67(9):2117–25. doi: 10.1007/s10840-024-01821-y (PMC11711855; doi:10.1007/s10840-024-01821-y)

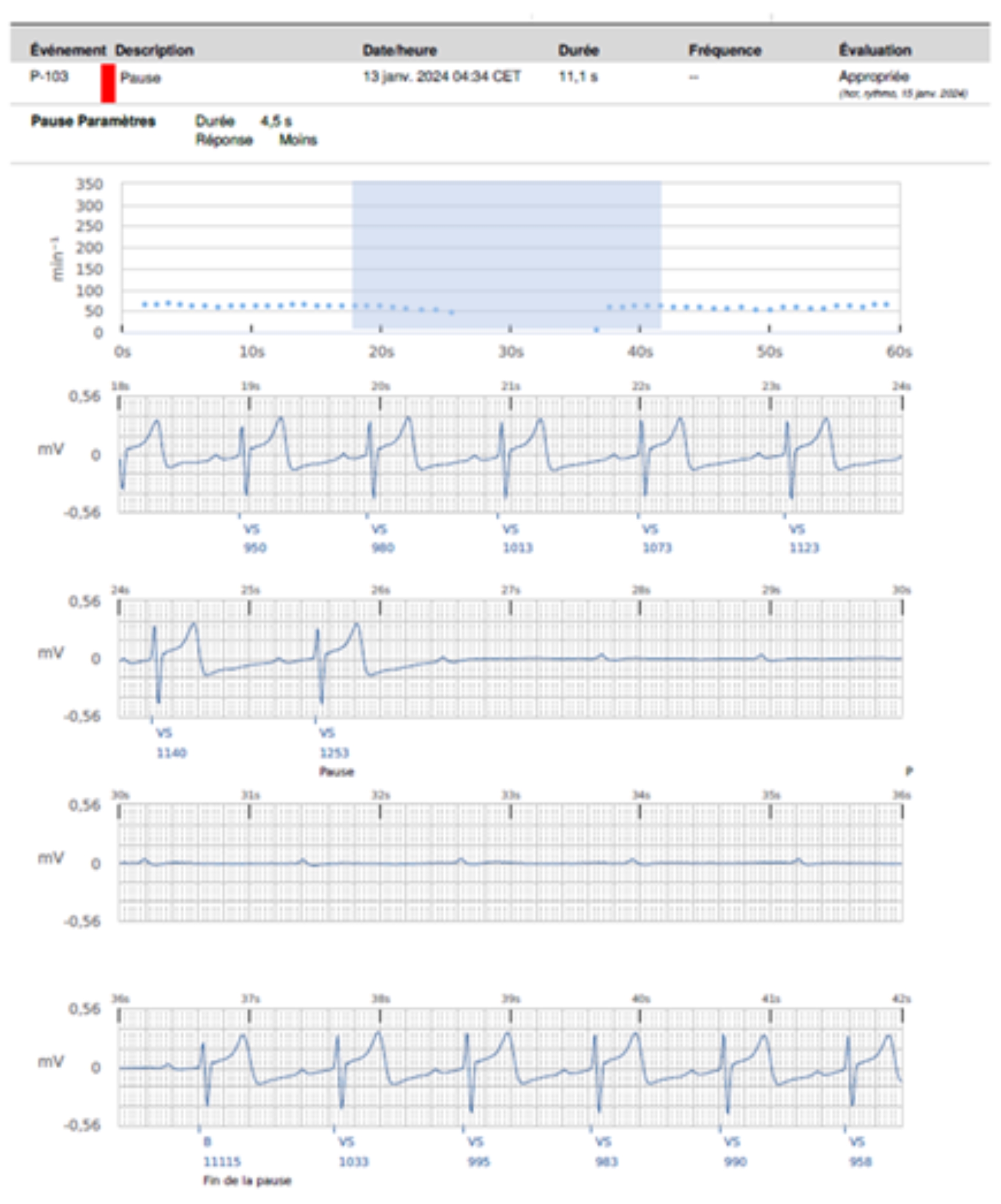

Supplement: Supplementary file 1 — Supplementary file1 (JPEG 762 KB) [file 10840_2024_1821_MOESM1_ESM.jpeg]

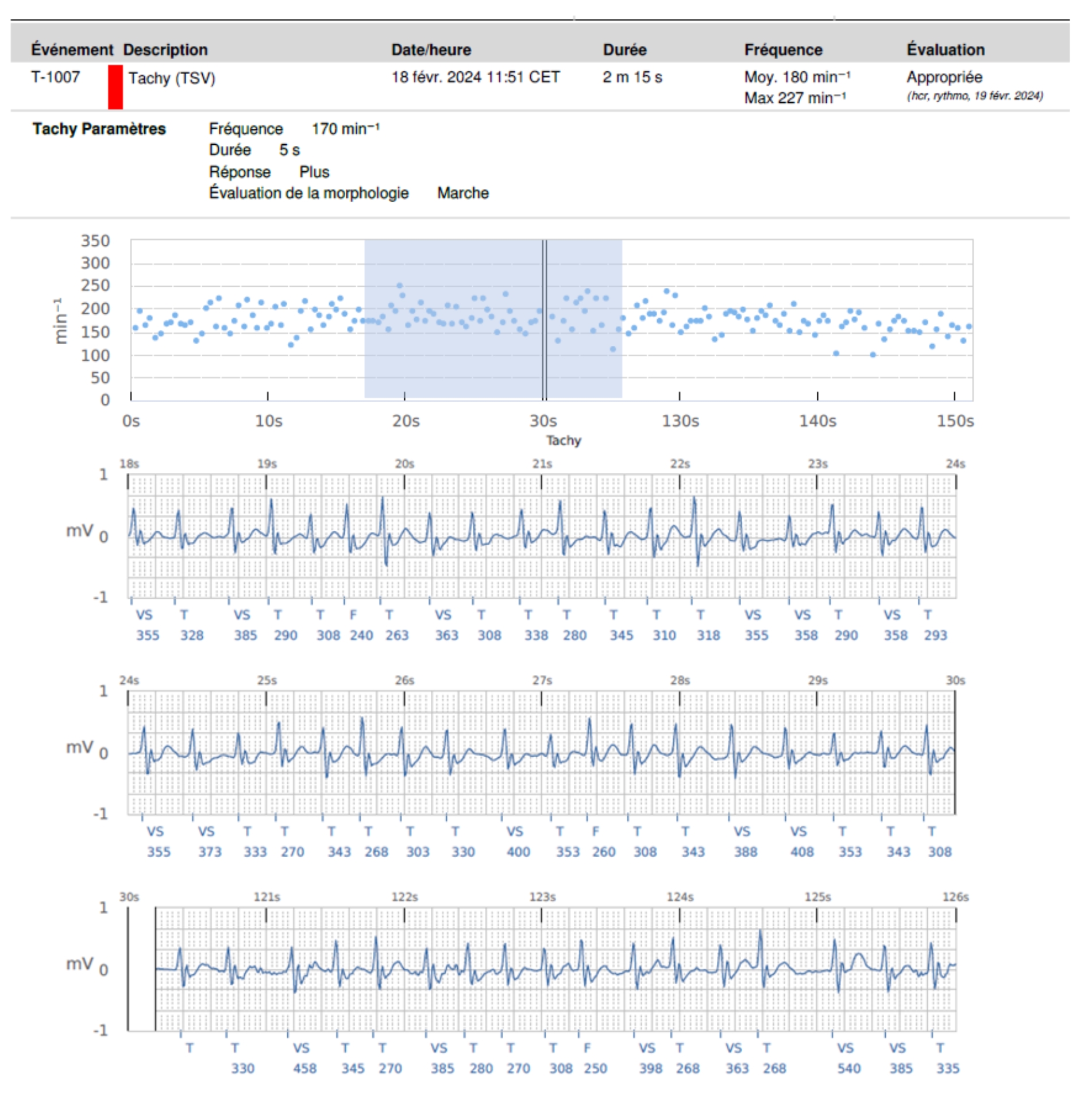

Supplement: Supplementary file 2 — Supplementary file2 (JPEG 982 KB) [file 10840_2024_1821_MOESM2_ESM.jpeg]

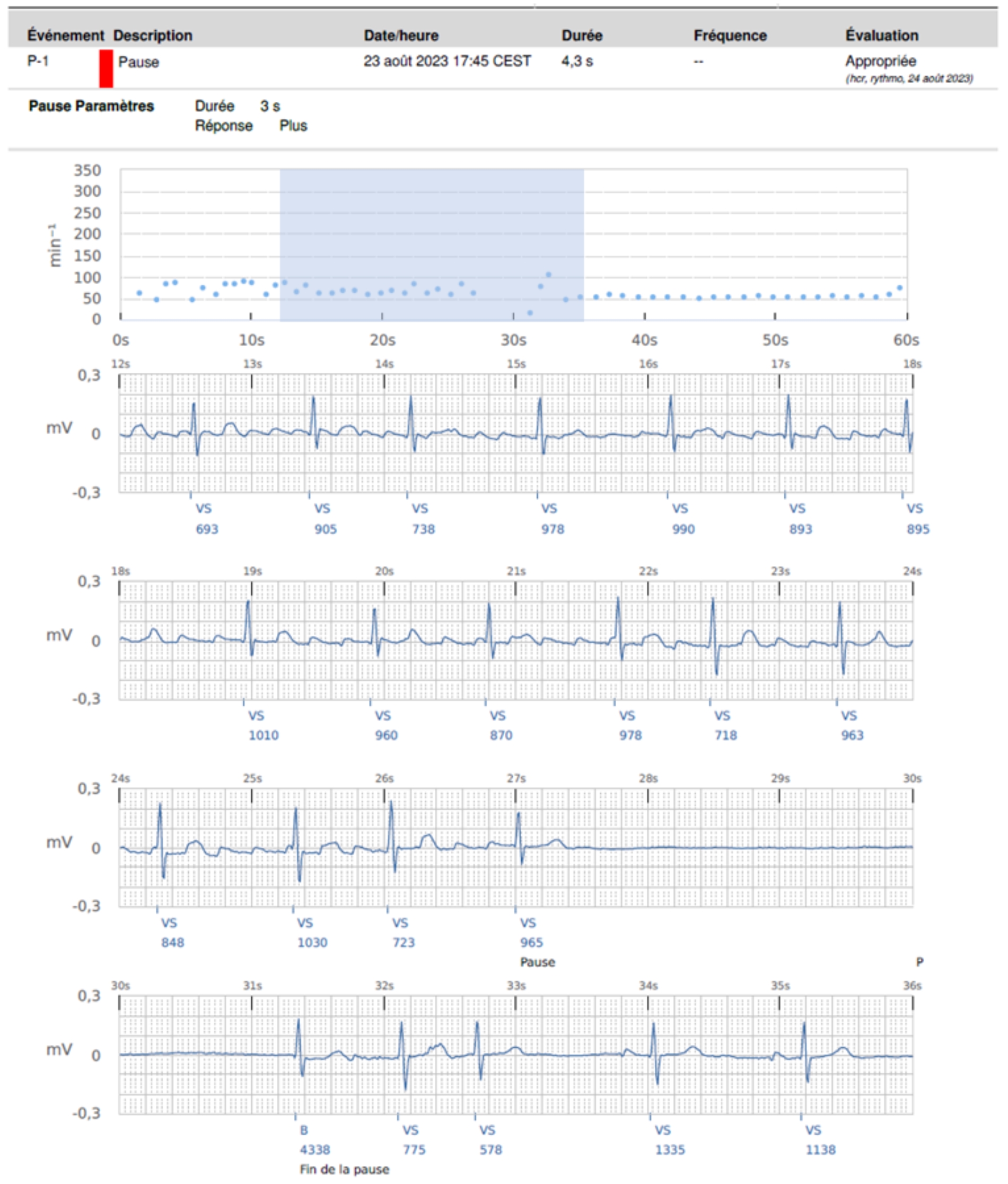

Supplement: Supplementary file 3 — Supplementary file3 (JPEG 1006 KB) [file 10840_2024_1821_MOESM3_ESM.jpeg]
